# Upregulation of *DWARF27* is associated with increased strigolactone levels under sulfur deficiency in rice

**DOI:** 10.1002/pld3.50

**Published:** 2018-04-11

**Authors:** Masato Shindo, Koichiro Shimomura, Shinjiro Yamaguchi, Mikihisa Umehara

**Affiliations:** ^1^ Graduate School of Life Sciences Toyo University Gunma Japan; ^2^ Graduate School of Life Sciences Tohoku University Sendai Japan

**Keywords:** *D27*, leaf senescence, macronutrient deficiency, *Oryza sativa*, shoot branching, strigolactones

## Abstract

Plants produce strigolactones (SLs) in roots in response to nitrogen or phosphate deficiency. To evaluate SL levels under other mineral deficiencies in rice, we cultivated rice seedlings in hydroponic media without nitrogen, phosphorus, potassium, sulfur, calcium, magnesium, and iron. Tiller bud outgrowth was stimulated under calcium deficiency because of low SL levels. SL levels increased under sulfur deficiency, in addition to phosphate, and nitrogen deficiencies. To explore which genes are key regulators of SL production under sulfur deficiency, we analyzed the expression of SL‐related genes in sulfur‐sufficient and sulfur‐deficient conditions. An SL biosynthesis gene, *DWARF27* (*D27*), was strongly expressed under sulfur deficiency, and its expression was decreased by sulfur supply. The levels of *D10*,* D17*, and *OsMAX1* transcripts did not differ between sulfur‐sufficient and sulfur‐deficient conditions. These results suggest that the increased SL levels under sulfur deficiency are due to a high expression of *D27*. A combination of nitrogen, phosphorus, and sulfur deficiencies had no additive synergistic effect on SL production. Under combined phosphorus and sulfur deficiency, the expression levels of most SL biosynthesis genes were elevated. The number of tiller buds in the *d27* mutant was higher than in the wild type, but lower than in other *d* mutants. Under sulfur deficiency, the chlorophyll content of *d27* was lower than those of other *d* mutants. These results indicate that *D27* plays an important role in adaptation to sulfur deficiency in rice.

## 1 INTRODUCTION

Strigolactones (SLs) are a class of terpenoid lactones that were originally discovered as stimulants of seed germination in root parasitic plants such as *Striga* spp., *Orobanche* spp., and *Phelipanche* spp. (for reviews, see Xie, Yoneyama, & Yoneyama, 2010; Yoneyama, Awad, Xie, Yoneyama, & Takeuchi, 2010). Later, SLs were found to induce hyphal branching of arbuscular mycorrhizal (AM) fungi, which support the acquisition of P and N in soil by the host plants (Akiyama, Matsuzaki, & Hayashi, 2005). Thus, SLs are thought to act as communication signals to recognize host plants for parasitism and symbiosis in the rhizosphere. In addition to the roles in the rhizosphere, SLs inhibit shoot branching (Gomez‐Roldan et al., 2008; Umehara et al., 2008). Because SL‐related mutants show pleiotropic phenotypes including enhanced shoot branching, SLs have recently been recognized as plant hormones that control plant growth at various developmental stages. In vascular plants, SLs stimulate stem thickening, leaf senescence, root hair elongation, and primary root growth and suppress adventitious root formation (Agusti et al., 2011; Kapulnik et al., 2011; Rasmussen et al., 2012; Ruyter‐Spira et al., 2011; Ueda & Kusaba, 2015; Yamada et al., 2014). In the moss *Physcomitrella patens*, SLs suppress chloronema branching and colony expansion (Proust et al., 2011).

**Figure 1:**
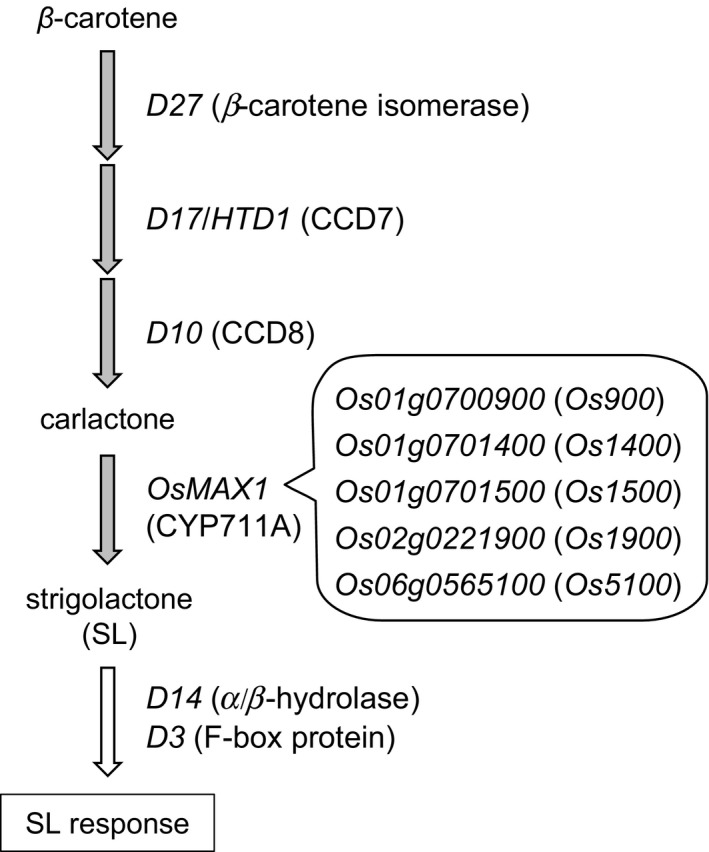
SL pathway in rice. Gray and white arrows indicate the SL biosynthesis and signaling, respectively

Carlactone (CL), a biosynthetic precursor of SLs, is synthesized from β‐carotene through consecutive reactions catalyzed by the β‐carotene isomerase DWARF27 (D27) and carotenoid cleavage dioxygenases 7 and 8 (CCD7 and CCD8) (Alder et al., 2012; Seto et al., 2014). In Arabidopsis, CL is converted to carlactonoic acid via oxidation by a cytochrome P450 encoded by *MORE AXILLARY GROWTH1* (*MAX1*) (Abe et al., 2014). In rice, *D17/HTD1* encodes CCD7, and *D10* encodes CCD8 (Arite et al., 2007; Zou et al., 2006), and there are five *MAX1* homologs: *Os01g0700900* (*Os900*), *Os01g0701400* (*Os1400*), *Os01g0701500* (*Os1500*), *Os02g0221900* (*Os1900*), and *Os06g0565100* (*Os5100*) (Nelson, Schuler, Paquette, Werck‐Reichhart, & Bak, 2004) (Figure 1). Os900 catalyzes the oxidation of CL into the parent SL 4‐deoxyorobanchol (4DO), and Os1400 catalyzes the hydroxylation of 4DO to orobanchol (Zhang et al., 2014). Canonical SLs have tricyclic lactone and methylbutenolide moieties connected by an enol ether bond. This bond and the methylbutenolide structure are essential for shoot branching inhibition (Boyer et al., 2012; Umehara et al., 2015). Configuration of the methylbutenolide moiety affects the biological activity of SLs in rice and Arabidopsis but not in pea (Boyer et al., 2012; Umehara et al., 2015). In SL signaling, D14 is a probable SL receptor and belongs to the α/β‐fold hydrolase superfamily, which also includes the gibberellin receptor GID1 (Arite et al., 2009) (Figure 1). D14 is transported through the phloem to tiller buds in rice (Kameoka et al., 2016). D3 is a leucine‐rich‐repeat F‐box protein that acts as a recognition subunit in a SKP1–CUL1–F‐box‐protein complex and binds target proteins for proteasomal degradation (Ishikawa et al., 2005) (Figure 1).

Several types of SLs have been identified; these are mainly produced in roots in response to P deficiency in various plant species including red clover, tomato, sorghum, *Lotus japonicus*, alfalfa, and rice (Lopez‐Raez et al., 2008; Sugimoto & Ueyama, 2008; Umehara et al., 2008; Yoneyama, Xie, et al., 2007; Yoneyama, Yoneyama, Takeuchi, & Sekimoto, 2007; Yoneyama et al., 2012). In rice, the levels of *D27*,* D17*,* D17*, and *Os900* mRNAs are highly elevated in roots under P deficiency and contribute to the high levels of SLs, indicating that most SL biosynthesis genes are upregulated in response to P deficiency (Umehara, Hanada, Magome, Takeda‐Kamiya, & Yamaguchi, 2010). Tiller bud outgrowth is not inhibited under P‐sufficient conditions because SL levels are low in roots. Under P deficiency, SL levels in roots are highly elevated and SLs are probably transported to tiller buds to inhibit the outgrowth. However, shoot branching is not suppressed under P deficiency in SL mutants of rice and Arabidopsis (Kohlen et al., 2011; Umehara et al., 2010). In some plant species such as lettuce, marigold, sorghum, and rice, SL levels in roots increase under both N deficiency and P deficiency (Sun et al., 2014; Yoneyama, Xie, et al., 2007; Yoneyama, Yoneyama, et al., 2007), and N and P fertilization suppresses SL production and exudation in roots (Umehara et al., 2010; Yoneyama, Xie, Kisugi, Nomura, & Yoneyama, 2013; Yoneyama et al., 2012). Thus, SLs are thought to be key regulators of efficient nutrient allocation and adaption to nutrient‐deficient environments (Umehara, 2011).

Little is known about how SL levels change in roots under deficiencies of macronutrients other than P and N. To address this question, here we examined the effects of deficiency in N, P, K, S, Ca, Mg, or Fe on SL production in rice. Here, we show that tiller buds of rice seedlings outgrow under Ca deficiency because SL levels decrease and that SL levels increase under S deficiency as well as N or P deficiency. We also show that *D27* is strongly expressed under S deficiency, and its expression is reduced by S supply, whereas the expression levels of *D10*,* D17*, and *OsMAX1* are not affected by S supply. These results suggest that SL production under S deficiency is due to a marked increase in the expression of *D27*. Under S deficiency, tiller bud outgrowth in a *d27* mutant was strongly suppressed compared with that in other *d* mutants, and chlorophyll content was lower than that in other *d* mutants, indicating that *D27* expression plays an important role in survival and adaptation to S deficiency.

## 2 MATERIALS AND METHODS

### 2.1 Plant materials and growth conditions

We used the rice (*Oryza sativa* L.) cultivar Shiokari as the WT and tillering dwarf mutants, *d10*,* d14*, and *d17* in the Shiokari background (Ishikawa et al., 2005). Seeds were provided by Dr. Junko Kyozuka (Tohoku University) and were propagated in a glass room at the Research Center for Life and Environmental Sciences, Toyo University. Seedlings were grown hydroponically as described previously (Umehara et al., 2008). Surface‐sterilized seeds were incubated in sterile water at 27°C in the dark for 1 day. Germinated seeds were put in hydroponic culture media solidified with 0.6% agar and cultured under fluorescent white light (130–180 μmol m^−2^ s^−1^; 16 hr light at 25°C, 8 hr dark at 23°C) for 7 days. The seedlings were then transferred to glass vials containing hydroponic culture medium (Kamachi, Yamaya, Mae, & Ojima, 1991). To evaluate the effect of nutrient deficiency on SL production, we prepared hydroponic culture media as shown in Table S1. All media contained 1 mM 2‐(*N*‐morpholino) ethanesulfonic acid (MES) buffer adjusted to pH 5.7. Only tillers growing over 2 mm were counted (Umehara et al., 2008). Relative chlorophyll contents were measured as SPAD values using a leaf chlorophyll meter, SPAD‐502Plus (Konica Minolta Inc., Japan) (Ata‐Ul‐Karim et al., 2016).

### 2.2 SL analysis

To measure the amounts of 4DO released from roots, hydroponic media were extracted with ethyl acetate twice after adding *d*_1_‐labeled 4DO as an internal standard. The ethyl acetate phase was evaporated to dryness under nitrogen gas. The extracts were dissolved in ethyl acetate: *n*‐hexane (15:85) and loaded onto 1‐ml Sep‐Pak Silica cartridges (Waters, MA, USA), washed with ethyl acetate: *n*‐hexane (15:85) and then eluted with ethyl acetate: *n*‐hexane (35:65).

To measure 4DO levels in roots, roots (ca. 650 mg) were homogenized in 10 ml acetone containing *d*_1_‐labeled 4DO; the homogenates were filtered with Bond Elute reservoirs (Agilent, CA, USA) and evaporated to dryness under nitrogen gas. The extracts were dissolved in water adjusted to pH 2–3 with 1 N HCl and extracted with ethyl acetate twice. The ethyl acetate phase was evaporated to dryness under nitrogen gas. The extracts were dissolved in 10% acetone, loaded onto Oasis HLB 3‐ml cartridges (Waters), washed with 10% acetone, and eluted with 60% acetone. The eluates were dissolved in ethyl acetate: *n*‐hexane (15:85), loaded onto Sep‐Pak Silica 1‐ml cartridges (Waters), washed with ethyl acetate: *n*‐hexane (15:85), and eluted with ethyl acetate: *n*‐hexane (35:65).

Purified 4DO‐containing fractions were dissolved in 50% acetonitrile and subjected to liquid chromatography–tandem mass spectrometry (LC‐MS/MS) analysis using a system consisting of a quadrupole tandem mass spectrometer (3200 QTRAP; Sciex, MA, USA) and a high‐performance liquid chromatograph (Prominence, Shimadzu, Kyoto, Japan) equipped with a reverse‐phase column (Acquity UPLC BEH‐C18, 2.1 × 50 mm, 1.7 μm, Waters). Data were analyzed in Analyst 1.5.1 and Multi Quant 2.0.2 (Sciex, MA, USA).

### 2.3 Gene expression analysis

Total RNA was extracted from roots using an RNeasy Maxi kit (Qiagen, Hilden, Germany) and concentrated using an RNeasy Mini kit (Qiagen). A 3‐μg aliquot of total RNA was used for cDNA synthesis with a ReverTra Ace qPCR RT Master Mix with gDNA Remover (Toyobo, Osaka, Japan). qRT‐PCR was performed on a StepOnePlus RT‐PCR system (Thermo Fisher Scientific, MA, USA) using a Thunderbird Probe qPCR Mix (Toyobo, Osaka, Japan), specific primers, and Taq‐Man probes listed in Table S2. Ubiquitin expression was used as internal control. Total RNA extraction, cDNA synthesis, and qRT‐PCR were conducted according to manufacturers' instructions.

### 2.4 Statistical analysis

Statistical analysis was performed in SPSS 23 (IBM SPSS Inc., Armonk, NY, USA) using Student's *t* test for pairwise comparisons, ANOVA and Tukey's honestly significant difference test (HSD) for multiple comparisons.

## 3 RESULTS

### 3.1 Effect of macronutrient deficiency in hydroponic culture medium on tiller bud outgrowth and SL production in rice

**Figure 2:**
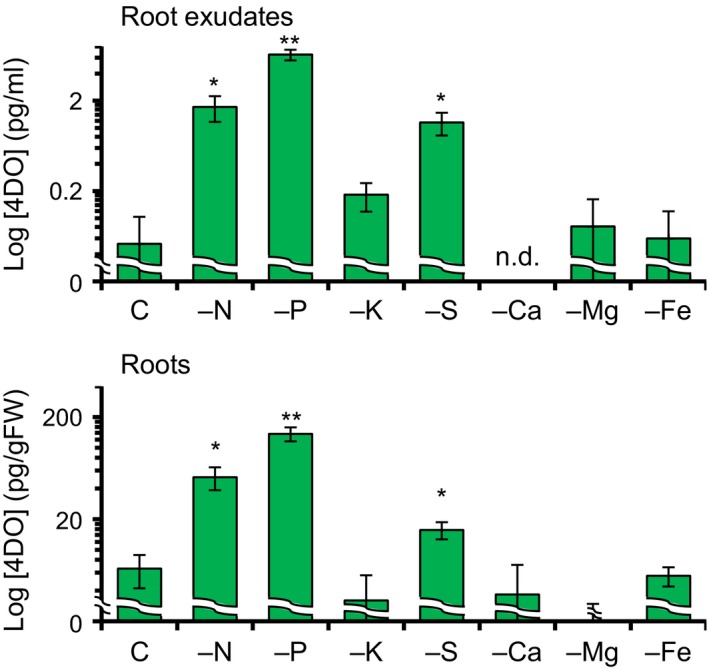
4DO levels in root exudates and roots under deficiency of N, P, K, S, Ca, Mg, or Fe. C, control. Samples were collected on day 7 after transfer to hydroponic media, and 4DO levels were analyzed using LC‐MS/MS. n.d., not detected. Data are means ± *SE* (*n* = 4). *p < .05, **p < .01 vs. control (Student's *t* test)

To evaluate the effect of macronutrient deficiency on tiller bud outgrowth in rice, we cultivated WT seedlings in hydroponic media without N, P, K, S, Ca, Mg, or Fe for 1 week and counted the number of outgrowing tiller buds (>2 mm). Interestingly, the second leaf tillers grew in the absence of Ca but not of other nutrients (Figure S1A). Tiller bud outgrowth in the absence of Ca was inhibited by 1 μM GR24 that is a SL synthetic analog (Figure S1B). LC‐MS/MS analysis demonstrated that the 4DO level was approximately 0.05 pg/ml in root exudates and 6.6 pg/g fresh weight (FW) in roots of nutrient‐sufficient control (Figure 2). Under Ca deficiency (–Ca), 4DO was not detected in root exudates and its content in roots (3.7 pg/g FW) was lower than that of control (Figure 2). The tiller bud outgrowth in –Ca plants was probably due to the reduction of 4DO levels in roots. Reduced SL levels under –Ca were reported in root exudates of red clover by liquid chromatography–tandem mass spectrometry (LC‐MS/MS) analysis and seed germination assay of root parasitic plants (Yoneyama, Xie, et al., 2007; Yoneyama, Yoneyama, et al., 2007).

Under –P conditions, the 4DO levels were highest among all experimental conditions tested (~6.5 pg/ml in root exudates and 137 pg/g FW in roots; Figure 2). Under –N conditions, the 4DO levels were also higher than in control (~1.7 pg/ml in root exudates and 51.5 pg/g FW in roots). Under –S conditions, the 4DO levels increased to ~1.1 pg/ml in root exudates and 15.7 pg/g FW in roots (Figure 2). To further investigate the effects of S deficiency on SL production, we grew rice seedlings for 1 week in hydroponic media with different S concentrations (potassium sulfate: 0, 10, 110, 210, and 310 μM as the S source). 4DO levels were higher at 0 and 10 μM than at 110, 210, and 310 μM potassium sulfate (Figure S2).

### 3.2 Time course analysis of SL levels and expression of SL‐related genes

**Figure 3:**
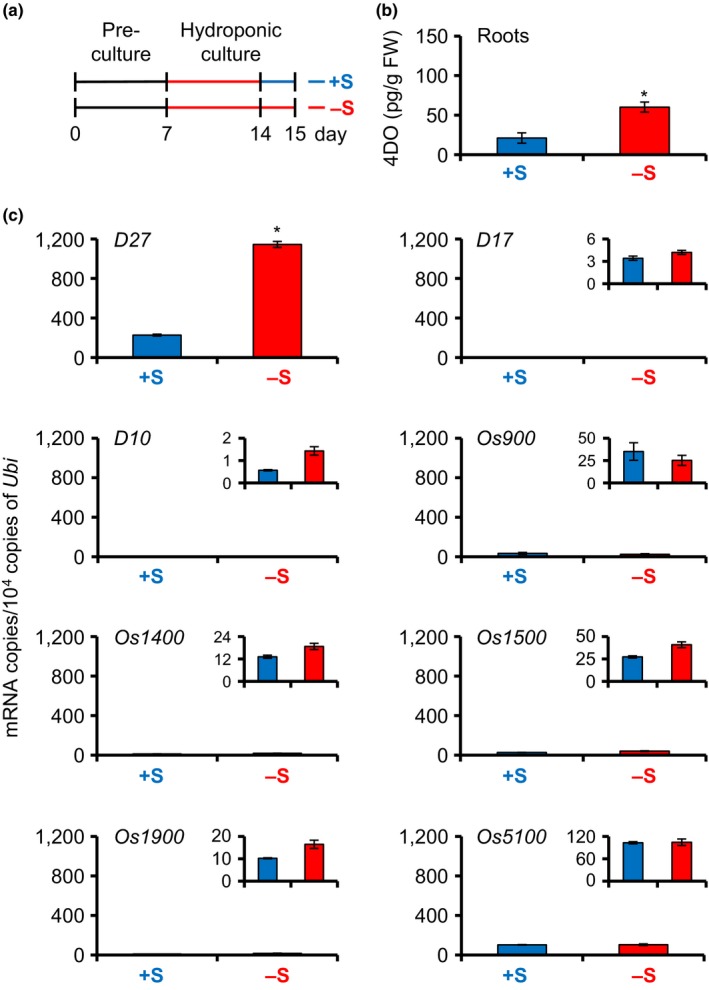
SL levels and expression of SL biosynthesis genes in WT rice seedlings under +S or −S conditions. (a) Schematic diagram showing the experimental conditions. Black and gray bars indicate +S and −S conditions, respectively. Seedlings were transferred to fresh +S or −S medium on day 14 of culture. 4DO levels and gene expression were analyzed on day 15. (b) 4DO levels in roots. Data are means ± *SE* (*n* = 4). (c) Transcript levels of SL biosynthesis genes in roots. Data are means ± *SE* (*n* = 3). *p < .05 (Student's *t* test)

To investigate which genes regulate SL production under S deficiency, we analyzed the expression levels of SL‐related genes using quantitative real‐time polymerase chain reaction (qRT‐PCR). We grew WT seedlings in hydroponic medium without S for 1 week, transferred them to fresh +S or −S medium, and analyzed 4DO levels and gene expression levels at 0, 1, and 2 days after transfer (Figures S3A). 4DO levels and expression of SL biosynthetic genes at 1 day were picked up in Figure 3. Although 4DO levels remained high under −S conditions, they were significantly reduced by S supply on days 1 and 2 after transfer (Figures 3b and S3B). *D27* was strongly expressed under −S conditions, and its expression was significantly reduced by S supply (Figures 3c and S3C). The expression pattern of *D27* resembled the changes in 4DO levels (Figure S3B,C). mRNA levels of *D10*,* D17*, and the five *MAX1* homologs did not differ significantly between +S and −S conditions (Figures 3c and S3C). On the other hand, the expression levels of the SL signaling genes *D3* and *D14* strongly increased under +S conditions in comparison with −S (Figure S3C).

### 3.3 SL production under N, P, and S deficiencies

**Figure 4:**
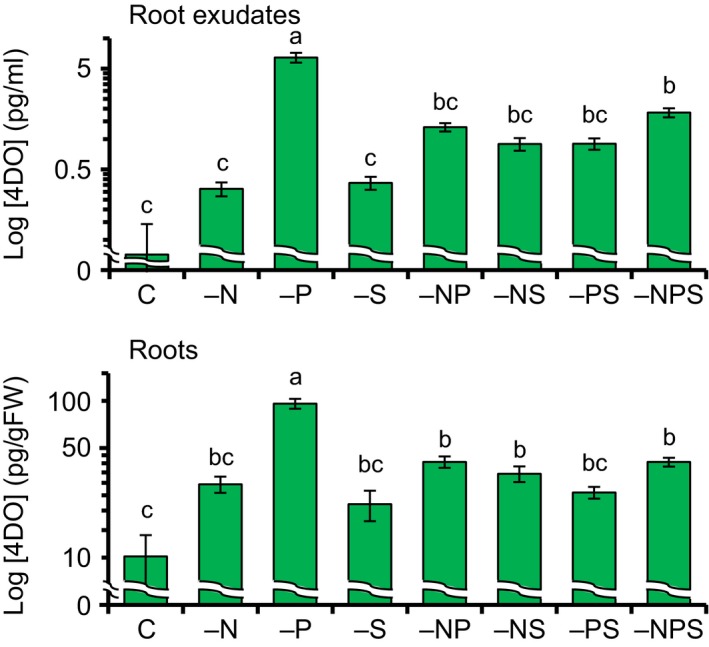
Effects of combined N, P, and S deficiencies on 4DO levels. The samples were collected on the day 7 after transfer to hydroponic culture media, and 4DO levels were analyzed using LC‐MS/MS. Data are means ± *SE* (*n* = 4). *p < .05, **p < .01 (Student's *t* test)

To investigate the effects of combinations of N, P, and S deficiencies on 4DO levels, we grew WT seedlings under nutrient‐sufficient conditions (control), single macronutrient deficiency, or combined deficiencies. 4DO levels increased significantly in all the treatments in comparison with control (Figure 4). The highest 4DO levels were observed in both root exudates and roots under P deficiency, but this increase was reduced in combinations of P deficiency with deficiency in N and/or S (Figure 4).

**Figure 5:**
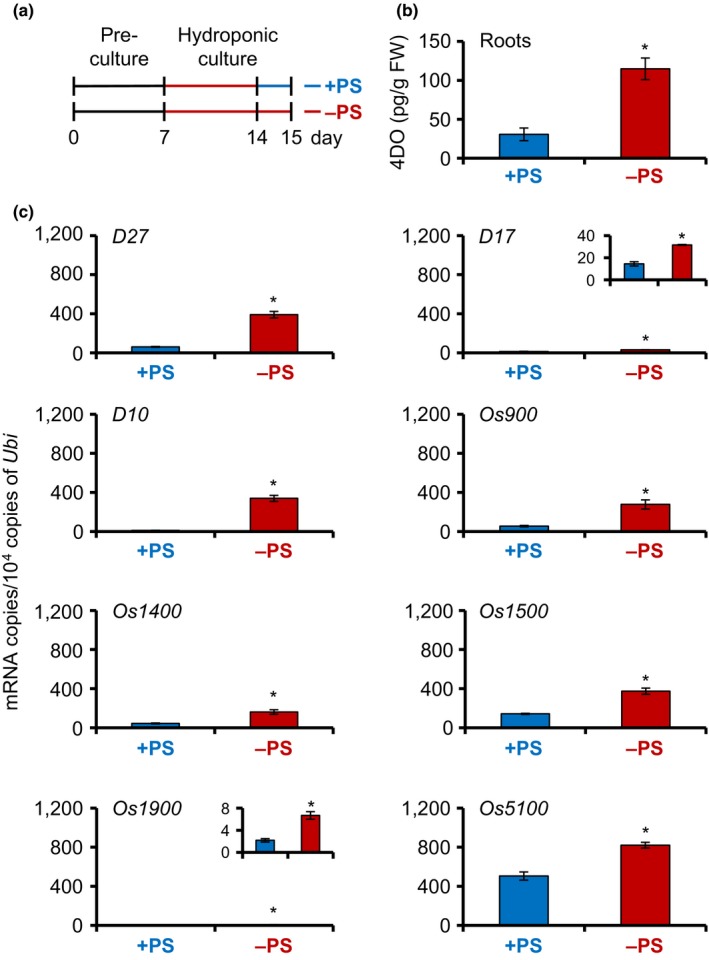
SL levels and expression of SL biosynthesis genes in WT rice seedlings under +PS or −PS conditions. (a) Schematic diagram showing the experimental conditions. Black and gray bars indicate +PS and −PS conditions, respectively. Seedlings were transferred to new +PS or −PS media on the day 14 of culture. 4DO levels and gene expression were analyzed on day 15. (b) 4DO levels in roots. Data are means ± *SE* (*n* = 4). (c) Transcript levels of SL biosynthesis genes in roots. Data are means ± *SE* (*n* = 3). *p < .05 (Student's *t* test)

To evaluate how the expression of SL biosynthesis genes changes in response to a combination of nutrient deficiencies, we analyzed the 4DO levels and expression of SL biosynthesis genes under combined P and S deficiency (−PS). We grew WT seedlings in −PS medium for 1 week and then transferred them to fresh +PS or −PS medium (Figure 5a). 4DO levels and gene expression were analyzed 1 day after the transfer. 4DO levels were slightly higher in the roots of seedlings grown in −PS medium than in those of seedlings grown in −S medium and were significantly decreased by PS supply (Figures 3b, 4, and 5b). *D27* expression was lower under −PS than under −S conditions, whereas the expression of other SL biosynthesis genes such as *D17*,* D10*, and *OsMAX1s* was higher (Figures 3c and 5c). On the other hand, the expression levels of the SL signaling genes *D3* and *D14* strongly increased under +PS conditions in comparison with −PS (Figure S4).

### 3.4 Effect of S deficiency on tiller bud outgrowth and leaf senescence

**Figure 6:**
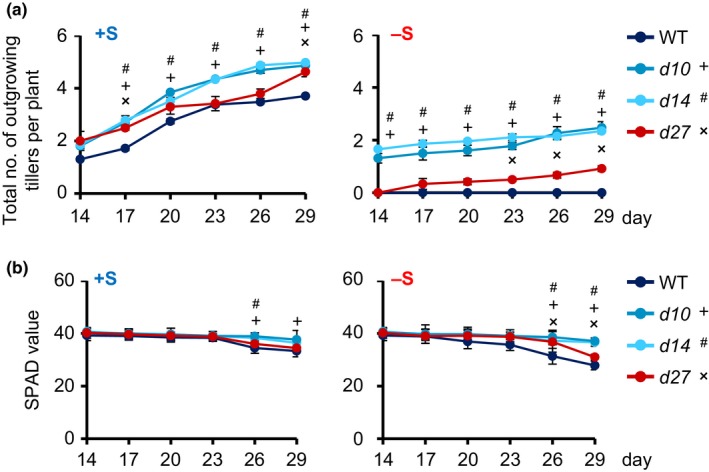
Phenotypical analysis of WT and SL mutants under +S and −S. (a) Total number of outgrowing tillers. Outgrowing tillers over 2 mm were counted in 21‐day‐old seedlings. Data are means ± *SE* (*n* = 3). (b) Comparison of the SPAD values of WT and the *d27*,* d10*, and *d14* mutants. Data are means ± *SE* (*n* = 3). Significant differences between WT and *d* mutants are shown (ANOVA,* p *< .05)

Shoot branching is suppressed and chlorosis is accelerated under S deficiency (Dobermann & Fairhurst, 2001). To evaluate whether shoot branching inhibition and chlorosis are caused by SL produced under S deficiency, we grew WT, the SL biosynthesis mutant *d10* and *d27*, and the SL signaling mutant *d14* in hydroponic culture medium with or without S for 22 days after preculture (Figure S5A). In WT, the number of outgrowing tillers over 2 mm gradually increased from 14 to 29 days under +S, but no tiller outgrowth was observed under −S (Figures 6a and S5B). In *d* mutants, the number of outgrowing tillers also gradually increased from 14 to 29 days under +S and was significantly reduced under −S (Figures 6a and S5B). The tiller bud outgrowth of *d27* was slower than those of other *d* mutants under +S, but achieved to similar levels at day 29 (Figure 6a). Under −S, the tiller number of *d27* was higher than that of WT but lower than those of other *d* mutants (Figure 6a).

Next, we pre‐cultured WT, *d10*,* d14,* and *d27* for 1 week, then grew them in hydroponic culture medium under +S for 1 week and then under −S or +S for 15 days (Figure S6A) and measured SPAD value in the third leaves (the SPAD value indicates total chlorophyll content, and low SPAD values indicate leaf senescence) (Ata‐Ul‐Karim et al., 2016). The SPAD value tended to gradually decrease with time and was significantly lower under −S than under +S from day 20 to day 29 in WT, and on day 29 in *d14* and *d27*, but did not differ significantly between +S and −S in *d10* (Figures 6b and S6B). The SPAD values were mostly similar from day 14 to day 29 between WT and *d* mutants under +S, but the SPAD values of WT were lower than *d* mutants on days 26 and 29 (Figures 6b and S6B). Interestingly, the SPAD values of *d27* were similar to WT and other *d* mutants under +S, but lower than that in other *d* mutants under −S (Figure 6b). To check a possibility that *d27* may produce slight levels of 4DO, we confirmed 4DO levels in *d27* using LC‐MS/MS. We could not detect 4DO in *d27* under either −S, −P, or nutrient‐sufficient conditions (Figure S7). The reduction in the number of outgrowing tiller buds and SPAD value in *d27* under −S was not observed under −P (Figure S8).

## 4 DISCUSSION

In this study, we found that 4DO levels increased under −S in addition to −P, and −N conditions in WT rice seedlings (Figure 2). Among SL biosynthesis genes, only *D27* (not *D10*,* D17*, or *OsMAX1s*) was highly expressed and thus contributed to SL production under −S (Figure 3). Thus, SL production under S deficiency is due to a marked increase in the expression of *D27*. In this time, we cannot exclude a possibility that activity of SL metabolism is low under −S because we still do not know the SL metabolic pathway. On the other hand, gene expression of *D3* and *D14*, which are the SL signaling genes, is rather upregulated under +S indicate that rice seedlings may increase the SL sensitivity because SL levels are low in S sufficient condition (Figures S3 and S4). When the *d27* mutant was grown under −S, it had fewer outgrowing tiller buds than other *d* mutants, and its chlorophyll content was lower than those of other *d* mutants (Figure 6). These results suggest that *D27* is involved in −S response and may maintain plant growth and development in −S conditions.

*D27* was originally characterized in rice as a gene encoding an iron‐containing protein that localizes in chloroplasts (Lin et al., 2009). *d27* mutants fail to produce SL, have increased tiller number and reduced plant height, and their phenotypes are rescued by exogenously applied SL, indicating that D27 is required for SL biosynthesis (Lin et al., 2009). In vitro experiments demonstrated that the D27 protein is an isomerase that converts all‐*trans*‐β‐carotene to 9‐*cis*‐β‐carotene in the first step of SL biosynthesis (Alder et al., 2012). In rice roots, high expression of most SL biosynthesis genes (*D27*,* D17*,* D10*, and *OsMAX1s*) increases SL levels under −P (Umehara et al., 2010). The expression levels of *D10* and *D17* increase under −N, but that of *D27* is not affected (Sun et al., 2014). In contrast, only *D27* is highly expressed under −S, and its level is 10 times that under −P (Umehara et al., 2010) (Figure 3). Under −PS, expression levels of *D27* decreased but that of the other SL biosynthetic genes increased compared with −S, resulting in increasing the flow of SL biosynthesis (Figures 3 and 5). Thus, 4DO content of PS deficiencies might be higher than that of S deficiency. D27 in Arabidopsis, AtD27, is located in plastids and acts upstream of MAX1 in the SL biosynthetic pathway (Waters, Brewer, Bussell, Smith, & Beveridge, 2012). Microarray data demonstrate that the expression level of *AtD27* in Arabidopsis roots increases under low S condition, but the expression of *MAX1* and *MAX3* do not (Iyer‐Pascuzzi et al., 2011; Maruyama‐Nakashita, Nakamura, Tohge, Saito, & Takahashi, 2006). These data support our results that *D27* expression is responsive to −S.

Sulfur is a macro‐element required for plant growth because it is a component of biologically important compounds such as the amino acids Cys and Met, antioxidant tripeptide glutathione, *S*‐adenosyl methionine, sulfolipids, and many secondary metabolites (Amtmann & Armengaud, 2009). In rice, S deficiency reduces chlorophyll content in young leaves, the number of tillers, and plant height (Dobermann & Fairhurst, 2001). These symptoms are similar to the phenotypes of SL biosynthesis‐ or signaling‐deficient mutants. In WT, tiller bud outgrowth was completely suppressed under −S, whereas tiller buds of *d* mutants still grew (Figure 6a). The SPAD values of *d10* and *d14* were higher than that of WT under −S (Figure 6b). Thus, SLs regulate shoot branching and leaf senescence in response to S deficiency. However, number of tiller buds and SPAD value of *d* mutants were smaller under −S than under +S, indicating that SLs only partially mediate the regulation of shoot branching and leaf senescence, and effect of nutrient deficiency are involved directly.

In SL biosynthesis, the transcription factors NSP1 and NSP2 are indispensable in *Medicago* and rice (Liu et al., 2011). *D27* expression is upregulated by NSP1 and NSP2 under −P and in rhizobium symbiotic signaling (Liu et al., 2011; van Zeijl et al., 2015). However, the expression levels of *NSP1* and *NSP2* did not increase in the absence of N or P (Liu et al., 2011). NSP1 and NSP2 were originally found as the GRAS family transcription factors regulating nodule formation (Kalo et al., 2005; Smit et al., 2005). Later, they were also found to be components of the Myc signaling pathway of AM fungi, in addition to the Nod signaling pathway (Delaux, Becard, & Combier, 2013; Lauressergues et al., 2012). In rice, only *D27* transcript increase in AM colonized roots compared with non‐AM colonized roots among SL biosynthetic genes (Kobae et al., 2018). Secreted SLs activate hyphal branching of AM fungi and trigger their symbiotic interactions with the host plants (Akiyama et al., 2005). AM fungi can supply S as well as N and P to the host (Allen & Shachar‐Hill, 2009; Govindarajulu et al., 2005; Smith & Read, 2008). Therefore, expression of rice *D27* is involved in −S response, and *D27* might play an important role in effective S absorption via AM fungi. To understand the interactions between SL production under −S condition and symbiosis with AM fungi, further research would be required in the future.

## CONFLICT OF INTERESTS

None declared.

## AUTHOR CONTRIBUTIONS

M.S. and M.U. performed the experiments and analyzed the data. K.S. and S.Y. contributed to the experimental design. M.U. directed the research and designed the experiments. M.S. and M.U. wrote the manuscript.
