# Supplementary material for: Upregulation of DWARF27 is associated with increased strigolactone levels under sulfur deficiency in rice
Source: Plant Direct. 2018 Apr 11;2(4):e00050. doi: 10.1002/pld3.50 (PMC6508544; doi:10.1002/pld3.50)
Supplement: Supplementary file 1 [file PLD3-2-e00050-s001.pdf]

Fig. S1

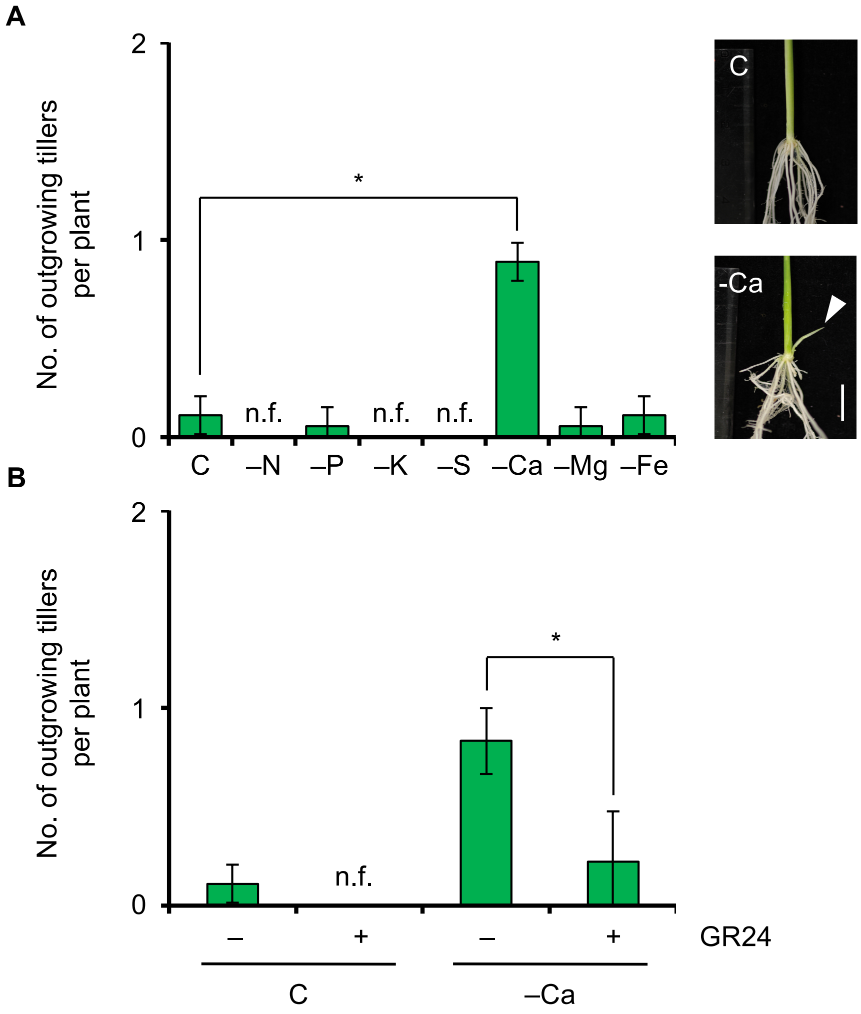

Figure S1 Effect of nutrient deficiency in rice. Wild-type seedlings were grown in hydroponic culture media without the indicated macronutrients for one week. C, control. Outgrowing tillers (>2 mm) were counted in 2-week-old seedlings. **A.** Effect of nutrient deficiency on tiller bud outgrowth. An arrowhead indicates the outgrowth of the second leaf tiller. n.f., not found. Scale bar = 1 cm. **B.** Complementation by 1 μM GR24. Data are means ± S.E. (n = 3). * $P < 0.05$ (Student's t-test).

Fig. S2

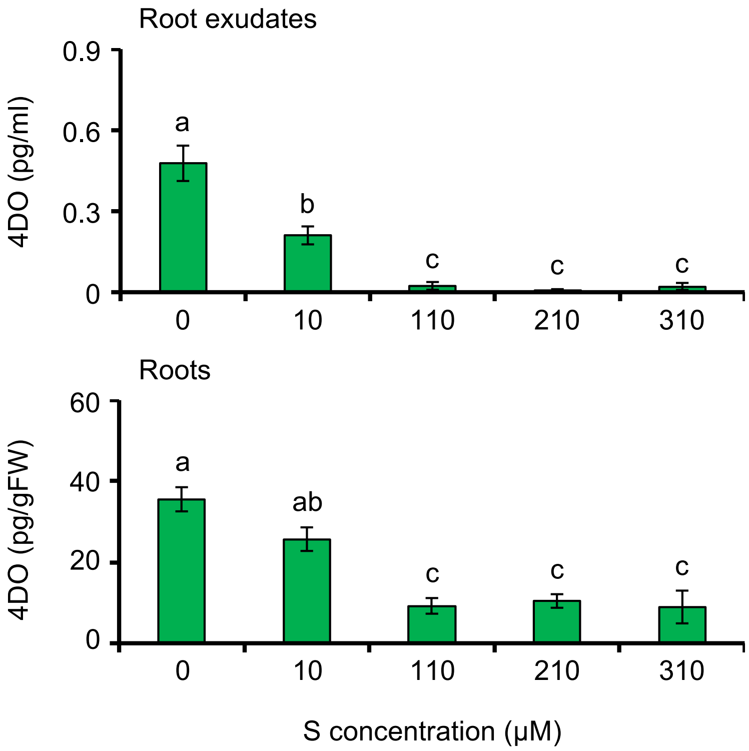

Figure S2 The levels of 4DO in root exudates and roots in WT rice seedlings at various S concentrations. Samples were collected on day 7 after transfer to hydroponic media, and 4DO levels were analyzed using LC-MS/MS. Data are means ± S.E. (n = 4). Different letters indicate significant differences (Tukey's HSD, $P < 0.05$).

Fig. S3

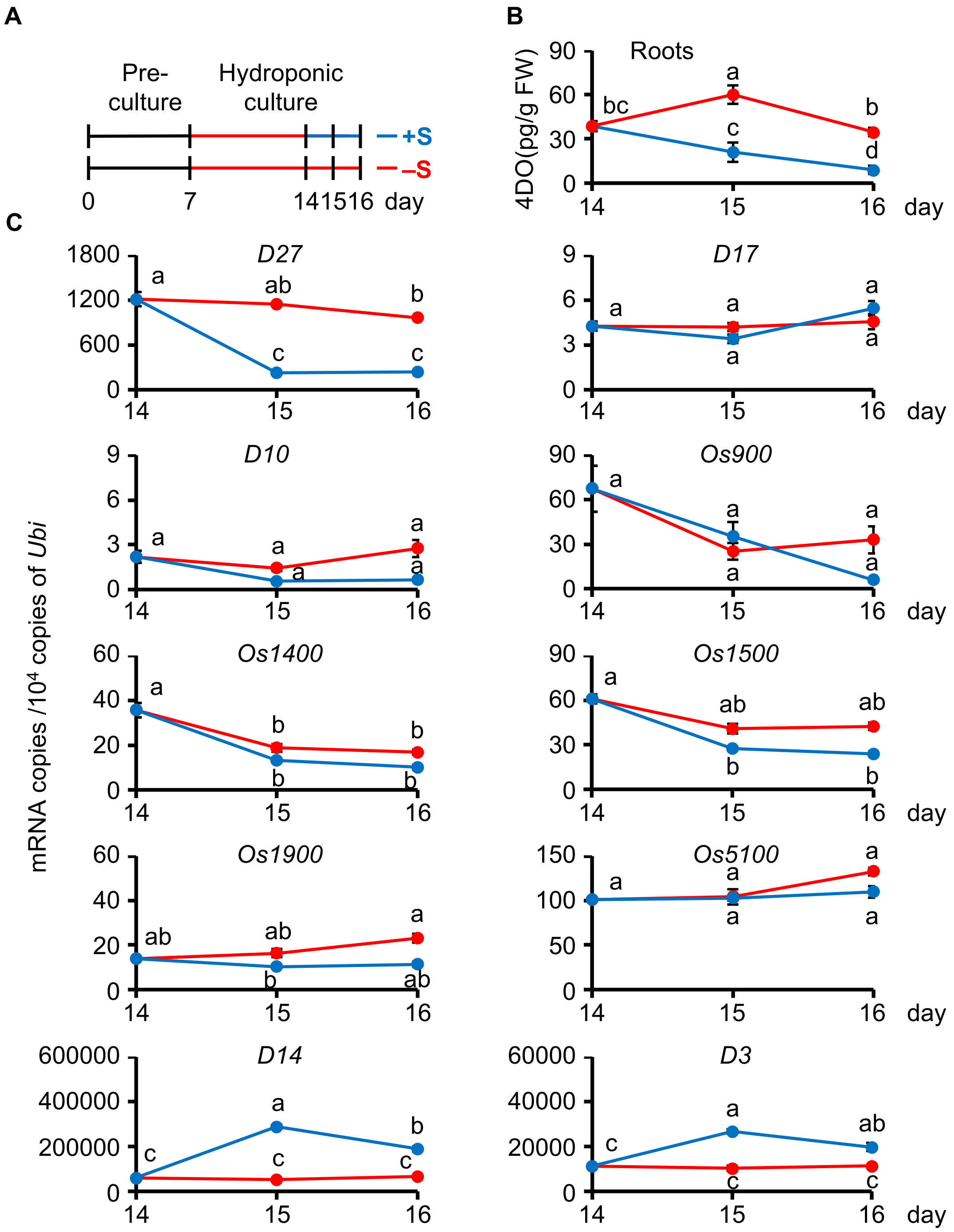

Figure S3 Time-course analysis of 4DO levels and expression of SL-related genes in WT rice seedlings. **A.** Schematic diagram of experimental conditions. Blue and red lines indicate +S and –S conditions, respectively. Seedlings were transferred to fresh +S or –S medium on day 14, and 4DO levels and gene expression were analyzed on days 14, 15, and 16. **B.** 4DO levels in roots. Data are means ± S.E. (n = 4). **C.** Transcript levels of SL-related genes in roots. Data are means ± S.E. (n = 3). Different lowercase letters indicate significant differences (Tukey's HSD, P < 0.05).

Fig. S4

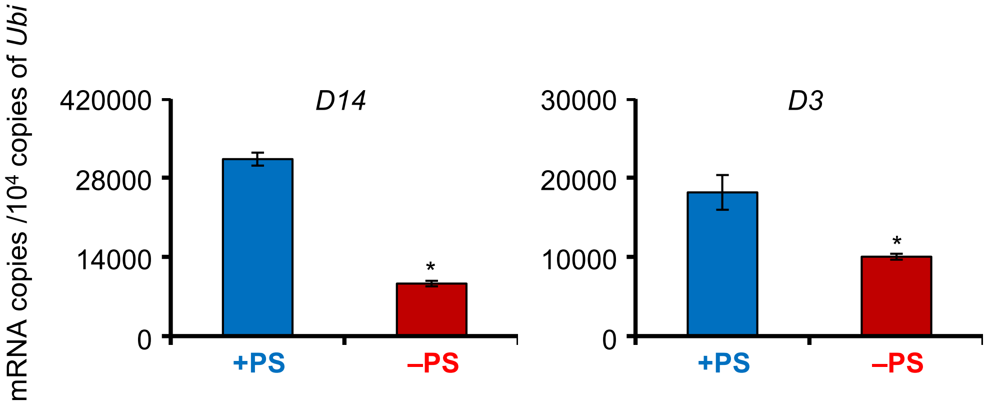

Figure S4 Transcript levels of the SL-signaling genes *D3* and *D14* in roots under +S and –S conditions. Data are means ± S.E. (n = 3). The experimental procedure is shown in Fig. 6A. Expression was analyzed on day 15 of culture. *P < 0.05 (t-test).

Fig. S5

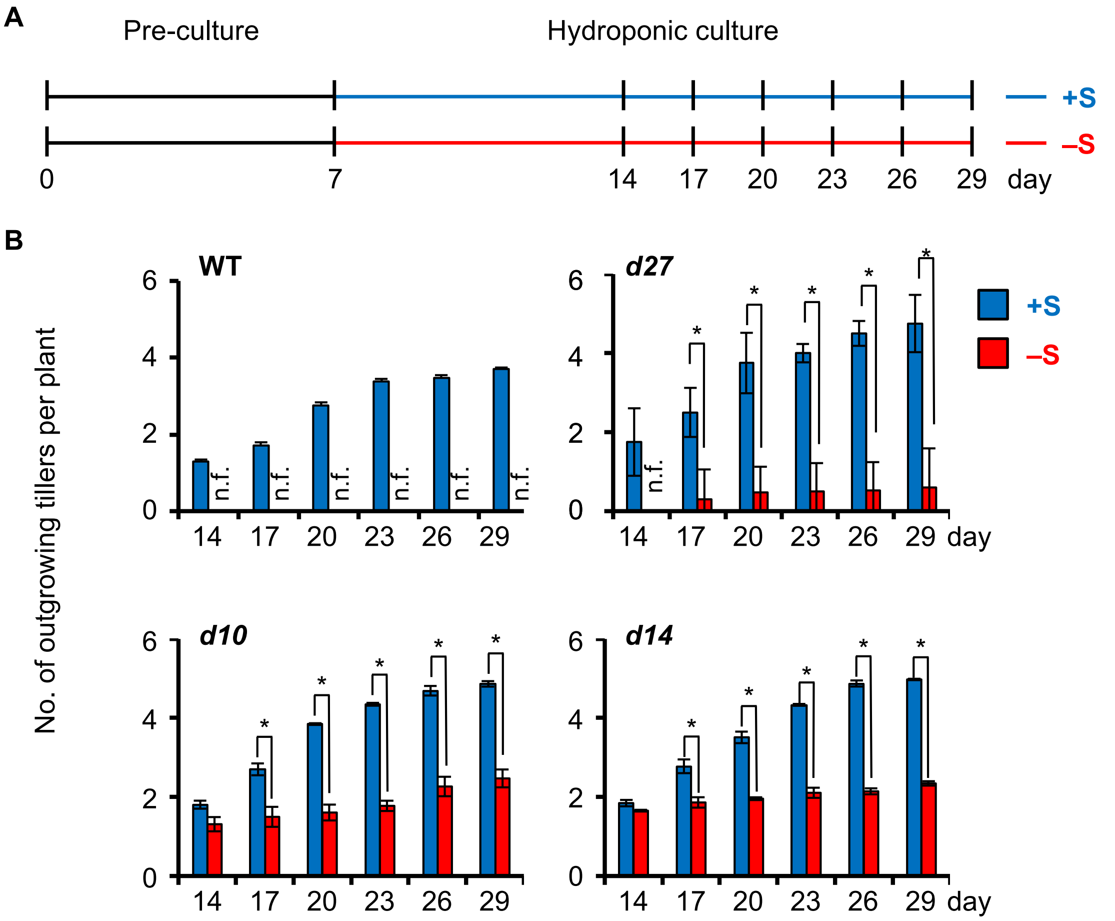

Figure S5 Effect of S deficiency on chlorophyll content. **A.** Schematic diagram of experimental conditions. Black and gray bars indicate +S and –S conditions, respectively. **B.** SPAD values indicating chlorophyll contents in the third leaves. Data are means ± S.E. (n = 3). * P < 0.01 (Student t-test). **C.** Comparison of the SPAD values of WT and the *d27*, *d10*, and *d14* mutants. Data are means ± S.E. (n = 3). Significant differences between WT and, *d10*, *d14* and *d27*are shown as +, #, × respectively (ANOVA, P < 0.05).

Fig. S6

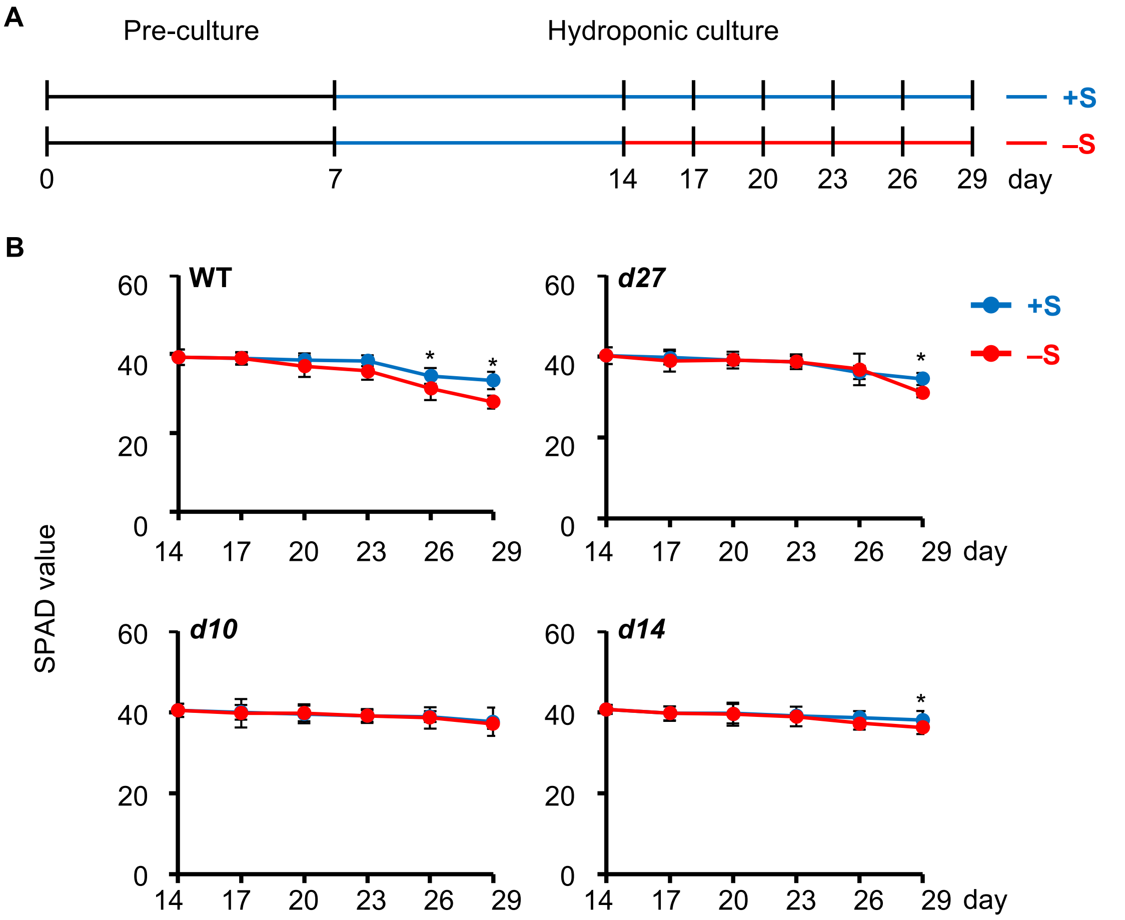

Figure S6 Effect of S deficiency on chlorophyll content. **A.** Schematic diagram of experimental conditions. Black and gray bars indicate +S and –S conditions, respectively. **B.** SPAD values indicating chlorophyll contents in the third leaves. Data are means ± S.E. (n = 3). * P < 0.01 (Student *t*-test). **C.** Comparison of the SPAD values of WT and the *d27*, *d10*, and *d14* mutants. Data are means ± S.E. (n = 3). Significant differences between WT and, *d10*, *d14* and *d27*are shown as +, #, × respectively (ANOVA, P < 0.05).

Fig. S7

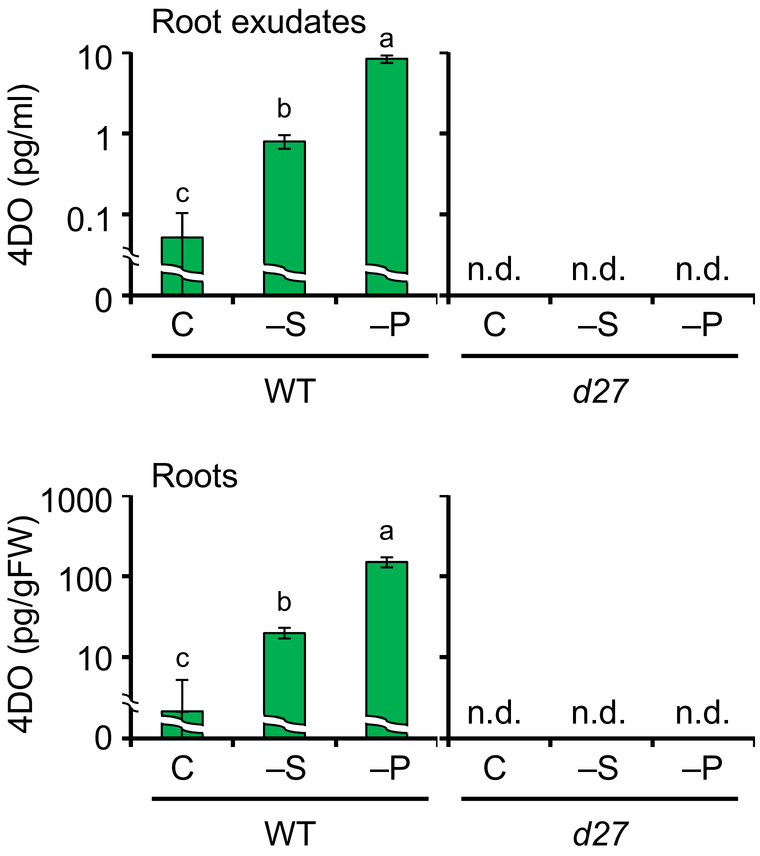

Figure S7 The levels of 4DO in root exudates and roots of WT and *d27* seedlings. C, control. Samples were collected on day 7 after transfer to hydroponic culture medium (C, –S, or –P) and 4DO levels were analyzed using LC-MS/MS. n.d., not detected. Data are means ± S.E. (n = 4). Different letters indicate significant differences (Tukey's HSD, $P < 0.05$).

Fig. S8

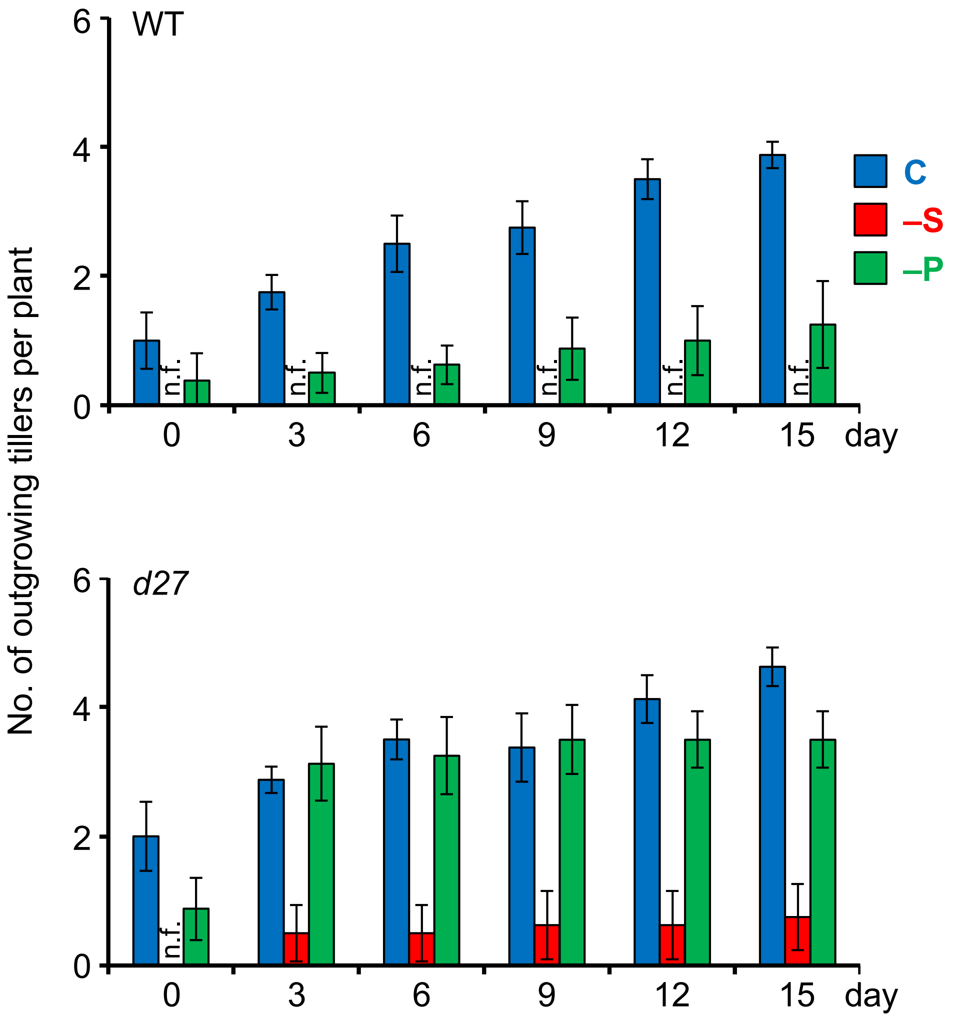

Figure S8 Effect of P or S deficiency on shoot branching. Outgrowing tillers over 2 mm were counted every 3 days after transfer to hydroponic culture media. C, control; n.f., not found. Data are mean ± S.D. (n = 8).
