# Supplementary material for: Upregulation of DWARF27 is associated with increased strigolactone levels under sulfur deficiency in rice
Source: Plant Direct. 2018 Apr 11;2(4):e00050. doi: 10.1002/pld3.50 (PMC6508544; doi:10.1002/pld3.50)
Supplement: Supplementary file 2 [file PLD3-2-e00050-s002.pdf]

Table S1 Hydroponic culture media used to investigate effects of nutrient deficiency.

Final concentration	Macro nutrient	Medium											
		C	–N	–P	–K	–S	–Ca	–Mg	–Fe	–NP	–NS	–PS	–NPS
1 mM	NH_4NO_3	+	-	+	+	+	+	+	+	-	-	+	-
0.6 mM	NaH_2PO_4	+	+	-	+	+	+	+	+	-	+	-	-
0.3 mM	K_2SO_4	+	+	+	-	-	+	+	+	+	-	-	-
0.3 mM	Na_2SO_4	-	-	-	+	-	-	-	-	-	-	-	-
0.3 mM	KCl	-	-	-	-	+	-	-	-	-	+	+	+
0.2 mM	$CaCl_2$	+	+	+	+	+	-	+	+	+	+	+	+
0.4 mM	$MgCl_2$	+	+	+	+	+	+	-	+	+	+	+	+
45 µM	Fe-EDTA	+	+	+	+	+	+	+	-	+	+	+	+
+S micronutrients*		+	+	+	+	-	+	+	+	+	-	-	-
-S micronutrients**		-	-	-	-	+	-	-	-	-	+	+	+

* 50 µM H_3BO_3, 9 µM $MnSO_4$, 0.3 µM $CuSO_4$, 0.7 µM $ZnSO_4$, and 0.1 µM $NaMoO_4$

** 50 µM H_3BO_3, 9 µM $MnCl_2$, 0.3 µM $CuCl_2$, 0.7 µM $ZnCl_2$, and 0.1 µM $NaMoO_4$

C, control; + supplied; – absent.

Table S2 Primers and TaqMan probes for qRT-PCR.

Primer*	5′-Sequence-3′
D3-F	CCCAACCTCCGCAAGCT
D3-R	GACGCAATCGCTGAACCG
D3-TaqMan	f-TGGCGCCATGCTTGTTCAACCC-t**
D10-F	CTGTACAAGTTCGAGTGGCACC
D10-R	CCTCGTCCGTCTCCTCGTAC
D10-TaqMan	f-CAAGGCCAGCGGCAAGATTG-t
D14-F	GCCTCTCCCCGGTTCTTG
D14-R	TGCTGTATCTCCTCCAGCTCG
D14-TaqMan	f-ACGACAGCGACTACCACGGCGG-t
D17-F	CCTCGTCCAGAAGCGTGAG
D17-R	TAGTGGGTGTCGGTGAAGGC
D17-TaqMan	f-TCGTCGTGCCGGACCACCTCA-t
D27-F	AGATGACCCTGCATTGAAGCA
D27-R	GCAATTCACACCATGATTCTGC
D27-TaqMan	f-CCATGCTTCCGGACAAAATGCG-t
Os5100-F	CTCTCCACCAGAAGGGCCTC
Os5100-R	GAGATGATCGTGTTCCTCATCG
Os5100-TaqMan	f-TCTTCACAAGGGACGCGAGGTGGT-t
Os900-F	CGTGAACCTCACGCTCGG
Os900-R	TTCATTGCAGCCGTCCG
Os900-TaqMan	f-CAGCTTGACAGGATCGTCGCCGA-t
Os1400-F	TGCATTGAGTGCGTGTCCA
Os1400-R	GAAGCCGAGAGCGAGATCG
Os1400-TaqMan	f-ACCTTGATGGCCAAGAGGACATAACATTCT-t
Os1500-F	AAGTGCTCAAGAGGATTCCCG
Os1500-R	CGATGCTGTCCATATGTGTTTTC
Os1500-TaqMan	f-CAAGATCGACCGGGTCAACCGC-t
Os1900-F	TACGTGGACGCTCTGGTGG
Os1900-R	TCTTGCCGATGATGTCGATG
Os1900-TaqMan	f-TCCCCTTCTGCCAGCTCTCGCTGT-t
Ubiquitin-F	AAGGTCACCAGGCTCAGGAAG
Ubiquitin-R	GATCGAAGTGGTTGGCCATG
Ubiquitin-TaqMan	f-CAACAACGACTGCGGCGCG-t

*F, forward primers; R, reverse primers.
**Fluorescent labels: f, FAM; t, TAMRA.

Figure S1 Effect of nutrient deficiency in rice. Wild-type seedlings were grown in hydroponic culture media without the indicated macronutrients for one week. C, control. Outgrowing tillers (>2 mm) were counted in 2-week-old seedlings. **A.** Effect of nutrient deficiency on tiller bud outgrowth. An arrowhead indicates the outgrowth of the second leaf tiller. n.f., not found. Scale bar = 1 cm. **B.** Complementation by 1 μM GR24. Data are means ± S.E. (n = 3). * $P < 0.05$ (Student's t-test).

Figure S2 The levels of 4DO in root exudates and roots in WT rice seedlings at various S concentrations. Samples were collected on day 7 after transfer to hydroponic media, and 4DO levels were analyzed using LC-MS/MS. Data are means ± S.E. (n = 4). Different letters indicate significant differences (Tukey's HSD, $P < 0.05$).

Figure S3 Time-course analysis of 4DO levels and expression of SL-related genes in WT rice seedlings. **A.** Schematic diagram of experimental conditions. Blue and red lines indicate +S and –S conditions, respectively. Seedlings were transferred to fresh +S or –S medium on day 14, and 4DO levels and gene expression were analyzed on days 14, 15, and 16. **B.** 4DO levels in roots. Data are means ± S.E. (n = 4). **C.** Transcript levels of SL-related genes in roots. Data are means ± S.E. (n = 3). Different lowercase letters indicate significant differences (Tukey's HSD, $P < 0.05$).

Figure S4 Transcript levels of the SL-signaling genes *D3* and *D14* in roots under +S and –S conditions. Data are means ± S.E. (n = 3). The experimental procedure is shown in Fig. 6A. Expression was analyzed on day 15 of culture. *$P < 0.05$ (t-test).

Figure S5 Effect of S deficiency on chlorophyll content. **A.** Schematic diagram of experimental conditions. Black and gray bars indicate +S and –S conditions, respectively. **B.** SPAD values indicating chlorophyll contents in the third leaves. Data are means ± S.E. (n = 3). * $P < 0.01$ (Student t-test). **C.** Comparison of the SPAD values of WT and the *d27*, *d10*, and *d14* mutants. Data are means ± S.E. (n = 3). Significant differences between WT and, *d10*, *d14* and *d27*are shown as +, #, × respectively (ANOVA, $P < 0.05$).

Figure S6 Effect of S deficiency on chlorophyll content. **A.** Schematic diagram of experimental conditions. Black and gray bars indicate +S and –S conditions, respectively. **B.** SPAD values indicating chlorophyll contents in the third leaves. Data are means ± S.E. (n = 3). * $P < 0.01$ (Student t-test). **C.** Comparison of the SPAD values of WT and the *d27*, *d10*, and *d14* mutants. Data are means ± S.E. (n = 3). Significant differences between WT and, *d10*, *d14* and *d27*are shown as +, #, × respectively (ANOVA, $P < 0.05$).

Figure S7 The levels of 4DO in root exudates and roots of WT and *d27* seedlings. C, control. Samples were collected on day 7 after transfer to hydroponic culture medium (C, –S, or –P) and 4DO levels were analyzed using LC-MS/MS. n.d., not detected. Data are means ± S.E. (n = 4). Different letters indicate significant differences (Tukey's HSD, $P < 0.05$).

Figure S8 Effect of P or S deficiency on shoot branching. Outgrowing tillers over 2 mm were counted every 3 days after transfer to hydroponic culture media. C, control; n.f., not found. Data are mean ± S.D. (n = 8).
