# Supplementary material for: Upregulation of DWARF27 is associated with increased strigolactone levels under sulfur deficiency in rice
Source: Plant Direct. 2018 Apr 11;2(4):e00050. doi: 10.1002/pld3.50 (PMC6508544; doi:10.1002/pld3.50)
Supplement: Supplementary file 3 [file PLD3-2-e00050-s003.pdf]

20 February 2018

Mikihisa Umehara
Graduate School of Life Sciences
Toyo University
1-1-1 Izumino, Itakura-machi,
Ora-gun, Gunma 374-0193, Japan
Tel. & Fax: +81-276-82-9144
E-mail: umehara@toyo.jp

Dr. Ivan Baxter
Editor-in-Chief
Plant Direct

Dear Dr. Ivan Baxter,

With this letter, I am submitting a revised version of our manuscript (MS No. 2018-00125-T1) entitled "**Up-regulation of *DWARF27* is associated with increased strigolactone levels under sulfur deficiency in rice**".

We thank the editors and the reviewers for their comments on our manuscript. We have revised it accordingly and highlighted the revised parts in yellow. We have also corrected some typographical errors that are not described in this letter and have highlighted these changes in yellow as well. Please find our detailed responses below.

This manuscript contains 6 figures and 8 supplemental figures.

We would greatly appreciate it if you could consider the revised version of our manuscript for publication in *Plant Direct*. We hope that our responses and the resulting changes are satisfactory, but we will be happy to work with you, the editors, and the reviewers to resolve any remaining issues. We look forward to your comments and those of the reviewers.

Sincerely,

Mikihisa Umehara (on behalf of all authors)

Responses to the reviewer’s comments

Reviewer 1

1. Consider modifying the title to be more representative of your conclusions. I would suggest something like, ' Up-regulation of DWARF27 transcript in sulfur deficient rice is associated with increased strigolactone levels'.

The title has changed to ‘Up-regulation of *DWARF27* is associated with increased strigolactone levels under sulfur deficiency in rice’ (See l.9-10).

2. Make sure your conclusions regarding D27 and SL levels reflect the suggestive nature of your findings and are not overstated.

According to the reviewer comment, we have revised the manuscript. We have moved some data to supplemental information or have deleted. In addition, most part of hypothetical model have been modified.

3. The possible link between D27 and Aux/IAA signaling and AM fungi (see Discussion on pg 8 and 9) is interesting but does not seem to be the focus of this study. I suggest removing most of this unsupported conjecture. The proposed model (line 272-279) is especially speculative without the Aux/IAA genes and no data on these genes presented in this study. I strongly recommend removing or heavily editing this material in the discussion.

According to the reviewer’s comment, the Aux/IAA-related description has been deleted.

4. Figure 1 (Effect of Nutrient deficiency on rice tiller number) could be moved to the supplement. This is not the main point of the study and, while important, does not add much to the main conclusions.

Fig. 1 has been moved to supplemental information.

5. Figure 8 panel A and B (Effect if S deficiency on chlorophyll content) could also be moved to the supplement or eliminated. Panel C could then be combined with Figure 7.

Experimental conditions in the Figs. 7 and 8 of old version are slightly different. Thus, Fig. 8C could not be simply combined with Fig. 7. In this version, Fig. 7C and Fig. 8C of old version have combined and newly shown as Fig. 6. The other data and experimental diagrams have been moved to supplemental information.

6. For all figures, the shorthand names given in the text (eg Os1400) should be explained in the figure captions where appropriate. I would prefer if proper gene identifier numbers were also given in the caption or used instead of the shorthand names.

According to the reviewer 2 comment, we have added the SL biosynthetic pathway with full number of gene ID *Os01g0701400* in Fig. 1. However, we would remain the shortened name such as *Os1400* in other figures because the name was used in other some papers that have been published.

7. In general, there are a lot of figure panels that convey very little information. I recommend combining as many figures as possible and moving redundant panels or panels with negative results to the supplement if possible.

According to the reviewer comment, figures have been re-organized. Fig. 1 has been moved to supplemental information. Fig. 3 has removed. Finally, fig. 7C and 8C have combined and the other data set have been moved to supplemental information

Reviewer 2

1. As you have lots of information about strigolactones biosynthesis, it will be much better to draw a model of SL biosynthetic pathway in planta. This would help readers understand present work easily.
The SL biosynthetic pathway has been shown in Fig. 1.

2. You demonstrated that sulfur deficiency significantly enhanced higher expression of D27 gene but not significantly for the other strigolactones biosynthesis genes. In other word, only D27 plays a key role in increasing SL levels in the biosynthetic pathway while others just have minors in this pathway under S-deficiency, is it right? Do you have any ideals to discuss it? This detailed discussion will strengthen the significance of present findings.
Yes, only D27 plays an important role in increasing SL levels under S deficiency among the SL-biosynthetic genes. The other SL biosynthetic genes have minor effect in SL production under S deficiency. *D27* is regulated by the transcription factors NSP1 and NSP2, which are components of the Myc signaling pathway of AM fungi. Secreted SLs activate hyphal branching of AM fungi and trigger their symbiotic interactions with the host plants. Only *D27* transcript increase in AM colonized roots. AM fungi can supply S as well as N and P to the host. Thus, *D27* might play an important role in effective S absorption via AM fungi (See l.291-306).

3. P5, L155 and L156, it seemed D10, Os900, Os1500, Os1900 expressed significantly different between +S and -S conditions at day 16? (Fig.S2C). You can check these data again.
We have checked the data of Fig. S2C. However, there are no significant difference between +S and -S on *D10*, *Os900*, *Os1500*, and *Os1900* expression in Tukey's HSD analysis.

4. P6, L157. Present work focused on the sulfur deficiency and strigolactones levels. I can not understand what the authors want to say for the results of D3 and D14 expression.
We have analyzed the SL-signaling genes *D3* and *D14* expression as well as expression of the SL biosynthetic genes. In S sufficient condition, expression levels of the SL biosynthetic genes are low, whereas *D3* and *D14* expression are rather up-regulated. These results indicate that rice seedlings may increase the SL sensitivity because SL levels are low in S sufficient condition. We have added these sentence in Discussion (See l.260-263).

5. P7 L194, the d27 mutant was not pre-cultured for these experiments?
No, the *d27* mutant was also pre-cultured in the experiment. We have added *d27* in the sentence (See l.230 and 239).

6. P8-P9, L255-L279. Although it is great to propose a hypothetical model of plant adaptation through SLs under -S, you have not any other data such as NSP1, NSP2,

Aux/IAA gene to support the model in your planta. I do not think lots of inference is good here

Our hypothetical model is just speculation in this time. Thus, most part of the model has been removed. However, NSP1 and NSP2 regulate *D27* expression and AM fungi can supply S to the host plant, provided by some papers. We would remain the description (See l.291-306).

7. P9 L288. You should check the mutant "d10 d14". This is s double mutant.

We are very sorry. A comma has been inserted between *d10* and *d14* (See l.116-117).

8. Check the format of literature cited. The journal "Plant Cell" should be replaced with "The Plant Cell".

'Plant Cell' has been changed to 'The Plant Cell'. In addition, 'Plant Journal' has been changed to 'The Plant Journal' (See REFERENCES).

9. It is interesting to investigate the effects of combinations of N, P and S deficiencies on 4DO levels. P-deficiency increased higher level of 4DO than S-deficiency. Is there any different mechanism to induce 4DO biosynthesis between P-deficiency and S-deficiency? However, combination of P-deficiency with S-deficiency induced less 4DO content than P-deficiency alone, but a bit more 4DO content than S-deficiency. Do you have any ideals to explain it?

We think there are different mechanism between P and S deficiencies because all SL biosynthetic genes are up-regulated under P deficiency (Umehara et al. 2010), whereas we found that only D27 is up-regulated under S deficiency in this study (See l.272-276). Up-regulating system is depending on the type of deficient macronutrient. Expression levels of *D27* decreased that of the other SL biosynthetic genes increased under PS deficiencies in comparison with S deficiency, increased the flow of SL biosynthesis. Thus, 4DO content of PS deficiencies might be higher than that of S deficiency (See l.276-279).

10. The Figs can be re-organized. For example, Fig. 1 and Fig. 2 can be combined together as one Figure.

Fig. 1 has been moved to supplemental information. We have deleted Fig. 3 of old version. In addition, Fig. 7C and 8C have been combined (now shown in new Fig. 6) and the other data and experimental diagrams have been moved to supplemental information.
